# Supporting the Medication Adherence of Older Mexican Adults Through External Cues Provided With Ambient Displays: Feasibility Randomized Controlled Trial

**DOI:** 10.2196/14680

**Published:** 2020-03-02

**Authors:** Ernesto Zárate-Bravo, Juan-Pablo García-Vázquez, Engracia Torres-Cervantes, Gisela Ponce, Ángel G Andrade, Maribel Valenzuela-Beltrán, Marcela D Rodríguez

**Affiliations:** 1 Facultad de Ingeniería Universidad Autónoma de Baja California Mexicali Mexico; 2 Facultad de Enfermería Universidad Autónoma de Baja California Mexicali Mexico

**Keywords:** health information systems, family caregiver, aged, medication adherence

## Abstract

**Background:**

Problems with prospective memory, which refers to the ability to remember future intentions, cause deficits in basic and instrumental activities of daily living, such as taking medications. Older adults show minimal deficits when they rely on mostly preserved and relatively automatic associative retrieval processes. On the basis of this, we propose to provide external cues to support the automatic retrieval of an intended action, that is, to take medicines. To reach this end, we developed the Medication Ambient Display (MAD), a system that unobtrusively presents relevant information (unless it requires the users’ attention) and uses different abstract modalities to provide external cues that enable older adults to easily take their medications on time and be aware of their medication adherence.

**Objective:**

This study aimed to assess the adoption and effect of external cues provided through ambient displays on medication adherence in older adults.

**Methods:**

A total of 16 older adults, who took at least three medications and had mild cognitive impairment, participated in the study. We conducted a 12-week feasibility study in which we used a mixed methods approach to collect qualitative and quantitative evidence. The study included baseline, intervention, and postintervention phases. Half of the participants were randomly allocated to the treatment group (n=8), and the other half was assigned to the control group (n=8). During the study phases, research assistants measured medication adherence weekly through the pill counting technique.

**Results:**

The treatment group improved their adherence behavior from 80.9% at baseline to 95.97% using the MAD in the intervention phase. This decreased to 76.71% in the postintervention phase when the MAD was no longer being used. Using a one-way repeated measures analysis of variance and a post hoc analysis using the Tukey honestly significant difference test, we identified a significant statistical difference between the preintervention and intervention phases (*P*=.02) and between the intervention and postintervention phases (*P*=.002). In addition, the medication adherence rate of the treatment group (95.97%) was greater than that of the control group (88.18%) during the intervention phase. Our qualitative results showed that the most useful cues were the auditory reminders, followed by the stylized representations of medication adherence. We also found that the MAD’s external cues not only improved older adults’ medication adherence but also mediated family caregivers’ involvement.

**Conclusions:**

The findings of this study demonstrate that using ambient modalities for implementing external cues is useful for drawing the attention of older adults to remind them to take medications and to provide immediate awareness on adherence behavior.

**Trial Registration:**

ClinicalTrials.gov NCT04289246; https://tinyurl.com/ufjcz97

## Introduction

### Background

One of the most common reasons for medication nonadherence among older adults is forgetfulness [1,2]. Problems with prospective memory, that is, the ability to remember future intentions, cause deficits in basic and instrumental activities of daily living, such as taking medications [3]. Consequently, the responsibility of managing medications of older adults and people living with dementia often falls on family caregivers [4-6]. Therefore, older adults need medication management technologies to help them not only with forgetfulness or cognitive impairment but also with other reasons that contribute to the lack of adherence to medications, such as polypharmacy (ie, the self-administration of multiple drugs) [2]. Our work is based on the facts that older adults show “substantial deficits when they depend on working memory and executive resources for prospective recall, but minimal deficits when they rely on associative recovery processes” [1]. Research has also shown that the use of external cues supports the automatic recovery of a planned action [1-10]. Therefore, we propose to support external cues through a tablet-based ambient display designed to increase the retrieval process of the planned action (ie, taking medications) and to provide awareness of adherence behavior [11,12]. To reach this end, we designed the Medication Ambient Display (MAD) [12,13].

An ambient display unobtrusively presents relevant information unless it requires the users’ attention [14]. In addition, users can easily monitor the display to obtain the desired information because it uses abstract modalities to represent information, such as pictures, sounds, and movement [14]. Thus, we used different abstract modalities to provide external cues that enable older adults to easily obtain relevant information to take their medications on time and be aware of their medication adherence.

### Previous Works and Study Rationale

In the last decade, different technological-based interventions for supporting the medication adherence of older adults have been studied. Owing to the rapid penetration of mobile phones, one of the approaches that has been widely studied is text message (SMS) reminders [15]. A systematic review identified that 18 of the 29 selected studies reported SMS-based interventions that improved older adults’ medication adherence [16]. A review of commercially available medication management apps for mobile phones reported that most of them focus on providing reminders [17]. A qualitative study identified that an interaction supported through linear navigation and multimodal reminder methods should be considered to increase the ease of use of mobile medication apps available on the internet; thus, these could be adopted by older adults [18]. Similarly, an adoption assessment of a commercial telehealth medication-dispensing device found that this is an acceptable tool for older adults to manage medications in collaboration with nurses [19].

Some research has been conducted to explore new computing approaches, such as ambient computing technologies, mobile games, and conversational agents specifically designed for older adults [20-23]. Several works have focused on supporting persuasive strategies to motivate older adults to follow their medication regimens. For instance, MoviPill is a mobile phone app that gamifies medication activity by awarding seniors who take their daily medication doses at the prescribed time and by promoting social competition [21]. dwellSense is a peripheral display that provides real-time and explicit feedback about medication-taking behaviors (eg, what medications were taken and whether they were taken on time, late, or not taken at all) [20]. This medication feedback encouraged seniors to reflect on their medication errors and then improve their medication self-efficacy [20]. Recently, the use of conversational agents accessible from mobile devices was explored to support educational strategies. The Conversational Medication Assistant for Heart Failure, named CARMIE, was developed to provide older adults with advice and information about their medication regimens, such as medication interactions, adverse reactions, and indications about how to take medications [23]. Similarly, the Electronic Medication Management Assistant is a chatbot developed to coach patients for managing medications prescribed by different health care providers, such as detecting double prescriptions, medication interactions, and contraindications [22]. In contrast to the previously mentioned works, our technological approach uses ambient modalities to provide external cues that aim to increase the retrieval process of the planned action and to provide daily and immediate awareness about how medication regimens are followed during the day. We assessed the effect of our approach by using objective medication adherence measures; moreover, we obtained qualitative findings that help us understand the adoption of the MAD. For this end, we provided seniors with the MAD to support the medication treatments prescribed by their physicians and the timetables that the seniors themselves proposed to follow.

### Objectives

Our study aimed to address the following research questions (RQ):

RQ1: What is the effect of the external cues provided by the MAD on older adults’ medication adherence?

RQ2: How do the MAD design features promote its adoption?

We used a mixed methods approach to obtain quantitative evidence regarding how several variables associated with adherence to medication improved and qualitative evidence regarding the adoption of the system by the participants. To describe our study, we used the suggestions by the Consolidated Standards of Reporting Trials to report pilot investigations [24] and electronic health interventions [25].

## Methods

**Figure 1:**
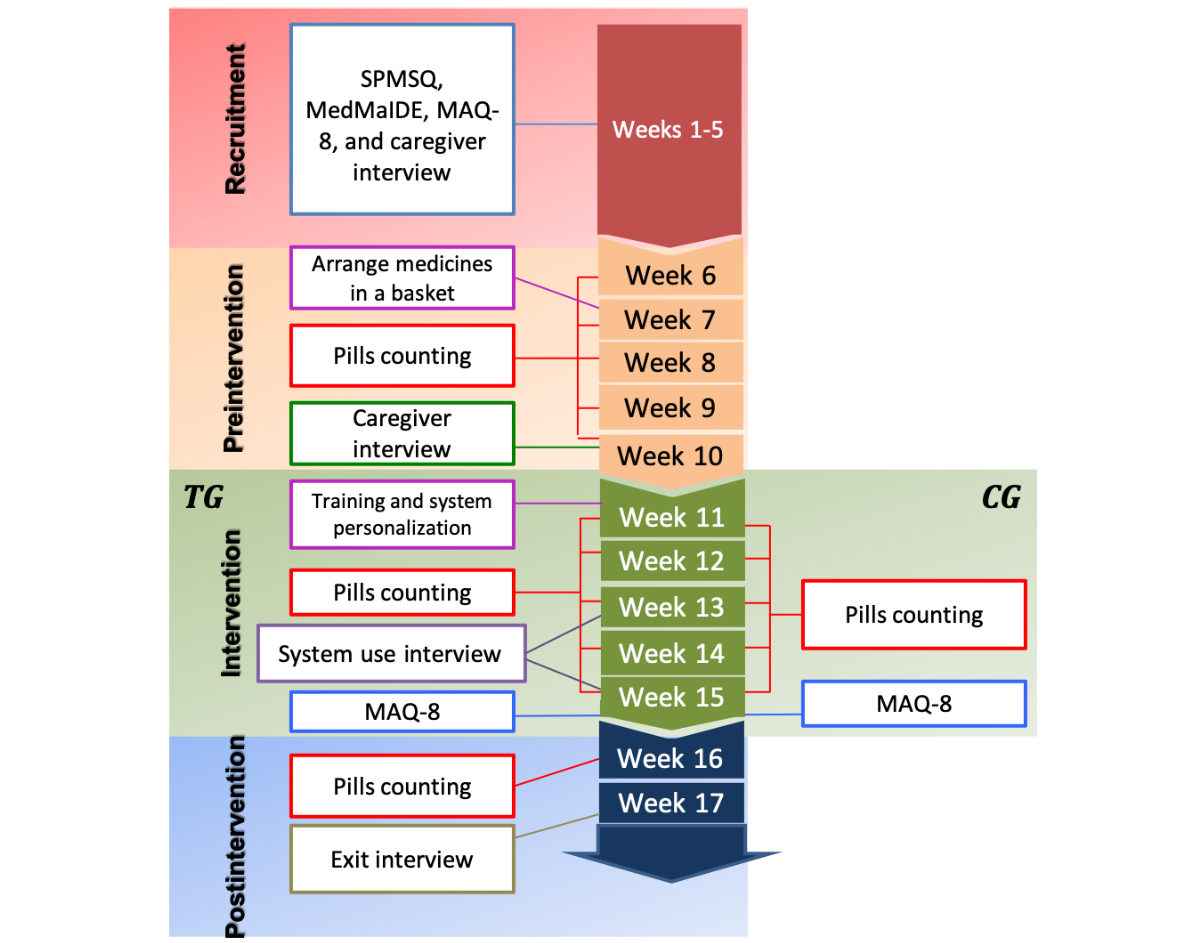
Study activities and instruments administered to participants during each study phase. CG: control group; MAQ-8: 8-item Medication Adherence Questionnaire; MedMaIDE: Medication Management Instrument for Deficiencies in the Elderly; SPMSQ: Short Portable Mental Status Questionnaire; TG: treatment group.

The evaluation of the MAD was designed as a small trial study. This section presents the study timeline, the activities conducted, and the instruments used to collect data (see Figure 1).

### Participants Recruitment

#### Eligibility Criteria

To be eligible, older adults had to meet the following criteria: be older than 60 years, take at least three medications prescribed by a physician (ie, polypharmacy), have mild cognitive impairment, report medication-forgetting events, and live with a relative who could provide us with information on the assistance required by the study participant to take their medications. The exclusion criteria were as follows: being unable to self-administer medications due to a functionality problem or severe cognitive impairment, and not taking pill-based medications (it may be difficult to assess adherence otherwise). To participate in the study, it was not a requirement that older adults have experience in the use of the internet or mobile devices.

#### Recruitment Procedure

The study was conducted in Mexicali, Mexico. Ten students from the Faculty of Nursing at the Universidad Autónoma de Baja California participated as research assistants. These students were enrolled in a social service program at the Community Center of the University (known as the UNICOM), which aims to provide seniors with occupational therapy and provide some health care assistance. The UNICOM is strategically located in a neighborhood where aging inhabitants predominate. For recruiting participants, research assistants contacted older adults in the vicinity of the UNICOM and administered a set of instruments as summarized in Table 1. First, the Spanish version of the 10-item Short Portable Mental Status Questionnaire was administered to detect the presence of mild cognitive impairment in the contacted seniors [26]. Then, the 8-item Medication Adherence Questionnaire (MAQ-8) scale was used to identify nonadherent seniors through 8 questions [27]. We selected this instrument because it is the quickest scale to administer, the simplest to score, and has been validated in many populations with different diseases and in persons with low literacy [28]. Finally, the Medication Management Instrument for Deficiencies in the Elderly (MedMaIDE) instrument was used to assess if the participants had deficiencies in managing medications and whether they met the polypharmacy criteria [29]. The MedMaIDE was designed to be administered in the home setting by nonclinical experts. It consists of 20 items to find out what patients know about their medications, whether they know how to take it, and how to get it from a doctor or pharmacy. During the MedMaIDE administration, relatives were permitted to participate to complement the seniors’ responses; this is considered appropriate to obtain a more reliable medication assessment [30]. In addition, relatives of older adults were interviewed to identify their role in helping older adults follow their medication routine. Older adults who met the eligibility criteria and expressed their interest to participate were enrolled in the study. The recruitment procedure lasted approximately 5 weeks.

**Table 1:** Instruments used to assess the eligibility criteria.

Eligibility criteria	Instrument	Score to be eligible
Mild cognitive impairment	Short Portable Mental State Questionnaire [26]	3 or 4 points
Medication deficiency	Medication Management Instrument for Deficiencies in the Elderly [29]	<13 points
Adherence for medicating	8-item Medication Adherence Questionnaire, also known as Morisky scale [27]	1-2 points=low and 3-8 points=medium
Caregiver involvement	Semistructured interview to find out how caregivers assisted older adults	—^a^

^a^Not applicable.

#### Organizational Setting

Once the participants were recruited, we realized that they were primarily of low socioeconomic status and affiliated with the Mexican Institute of Social Security (IMSS), the largest medical institution in Mexico. Periodically (monthly or bimonthly), they attended an IMSS clinic for follow-up consultation and to retrieve an updated prescription to get their medications from the clinic’s pharmacy. The lack of adequate health care and pharmaceutical policies to rationally manage medications and monitor the treatment of patients increases the vulnerability of Mexican seniors to medication errors [31], a situation that is also faced in other countries [32,33].

### Preintervention

**Figure 2:**
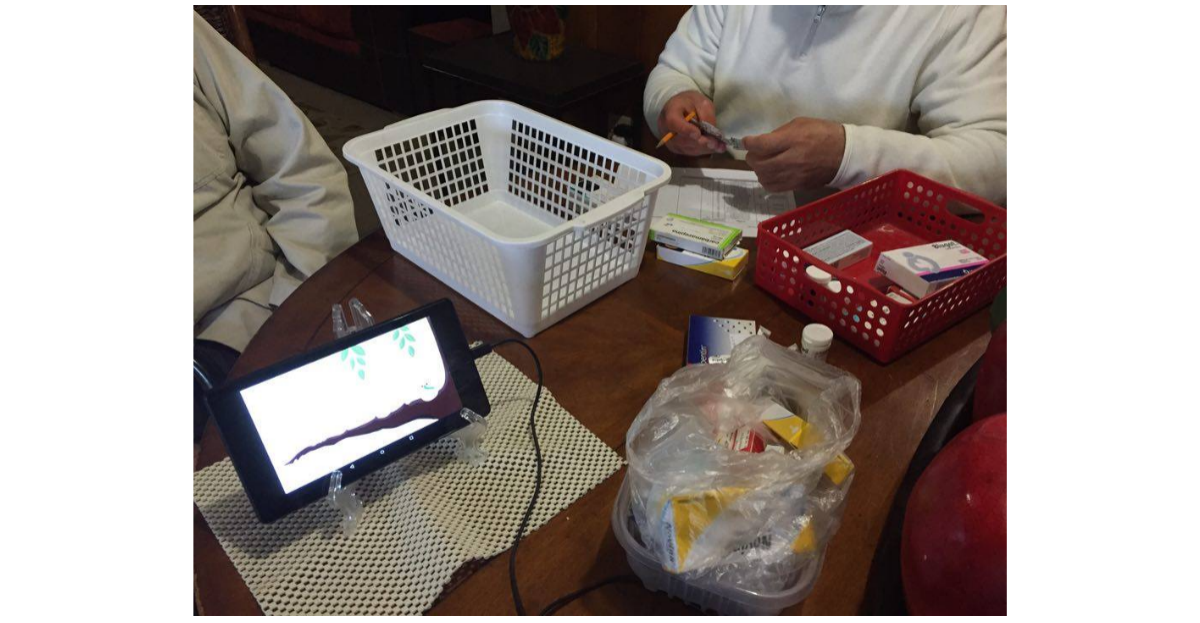
A research assistant arranging medications in a basket.

Baseline data were collected during weeks 6 to 10 on medication adherence by using the pill counting technique. We noticed that participants accumulated containers with the same medications. Under those circumstances, we provided seniors with a basket to arrange the medications that should be taken each week (see Figure 2), which facilitated data collection for measuring the *Dosage_pill_* adherence outcome (see Table 2).

**Table 2:** Outcome variables and methods used to collect data to address research question 1.

Variable	Description	Collection method
*Dosage_pill_* ^a^	The number of pills taken by participants in a period divided by the number of pills expected to be taken for that period^a^	Pill counting
*Dosage_MAD_* ^b^	The number of medication episodes reported as taken by participants in a period divided by the number of episodes expected to be recorded for that period	MAD’s^c^ log
Timely^d^	Indicates whether the medication was taken 30 min before or after the time expected to take the medication. This is the number of medication episodes registered in the time window during a period divided by the number of episodes registered as taken for that period	MAD’s log
Self-reported medication adherence^e^	A score estimated based on reported nonadherent behaviors; for example, drug omissions, medication forgetting, carelessness, or stopping a medication when feeling worse [28].	8-item Medication Adherence Questionnaire

^a^It measures whether the medication is not being taken as prescribed, which may affect the clinical outcome. We estimated it for both groups (treatment group and control group).

^b^It enabled us to understand how much older adults used the MAD reminders. It was estimated for the treatment group.

^c^MAD: Medication Ambient Display.

^d^It measures whether doses were taken during the prescribed interval. It was estimated for the treatment group.

^e^It is an assessment instrument to identify individuals’ perception about their medication adherence. The 8-item Medication Adherence Questionnaire was administered during the recruitment phase and at the end of the intervention phase.

### Intervention

#### Design and Implementation of the Medication Ambient Display

For medical information systems to be more specific to the needs of users, an iterative approach must be followed, which consists of different usability studies [34]. In this sense, our previous work included usability studies that helped us inform the design of the MAD. They included (1) a usability inspection in which experts (usability engineers and geriatricians) determined how the MAD conformed to usability design principles for ambient displays [11] and (2) a field evaluation to identify technical and usability problems that we addressed without altering the conceptual design of the MAD [12,13]. In this paper, we present the trial study that we conducted to evaluate the effect of the MAD on measures of adherence to medication and its possible adoption.

We implemented the MAD for Android tablets to be placed as portrait frames in the older adults’ homes and to provide the following cues:

- Abstract and stylized representations of their medication adherence
- Auditory and visual reminders to call older adults’ attention
- Events that may enhance older adults’ awareness about whether the medication was taken

#### Abstract and Stylized Representations of Medication Adherence

**Figure 3:**
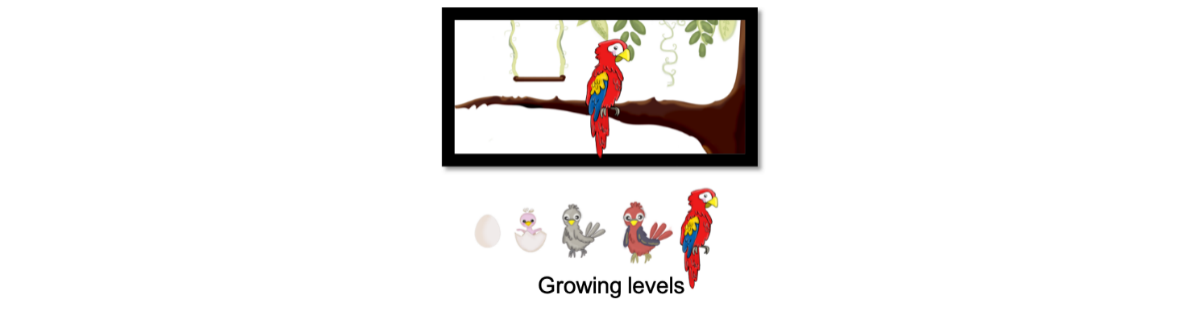
Abstract representations of medication adherence based on parakeet growth.

**Figure 4:**
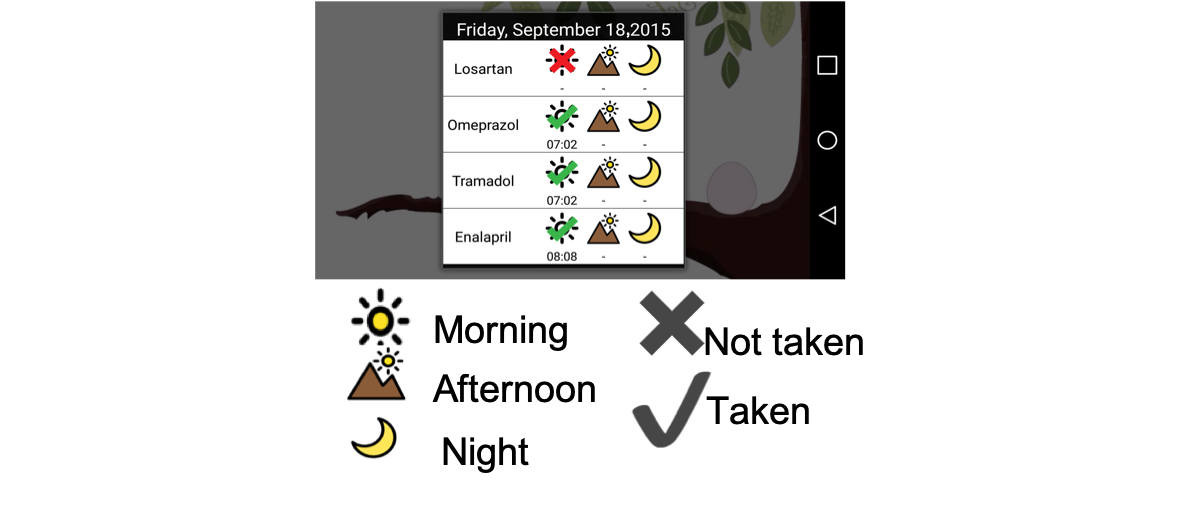
Detailed information about the medication adherence corresponding to the current day and notation used to represent if medicines were taken on time.

The MAD shows a virtual birdcage, which has the aim of raising elders’ consciousness about how they have to take responsibility for caring for their health, in a way similar to willingly caring for a pet. As presented in Figure 3, the abstract representation is an animation of a parakeet that symbolizes daily medication compliance. Each day, a newborn pet grows to represent medication compliance. In addition, by touching any point on the virtual cage of the parakeet, the MAD presents detailed information on the individual’s daily medication compliance by using the notation presented at the bottom of Figure 4. In this figure, the MAD shows that an older adult has to take 4 medicines, and each of them should be taken 3 times during the day: morning, afternoon, and night. Thus, it presents that a morning medicine (Losartan) was not taken and that the afternoon and night doses are still pending. Optionally, participants can consult their medication adherence from any other date.

#### Auditory and Visual Reminders to Call Older Adults’ Attention

**Figure 5:**
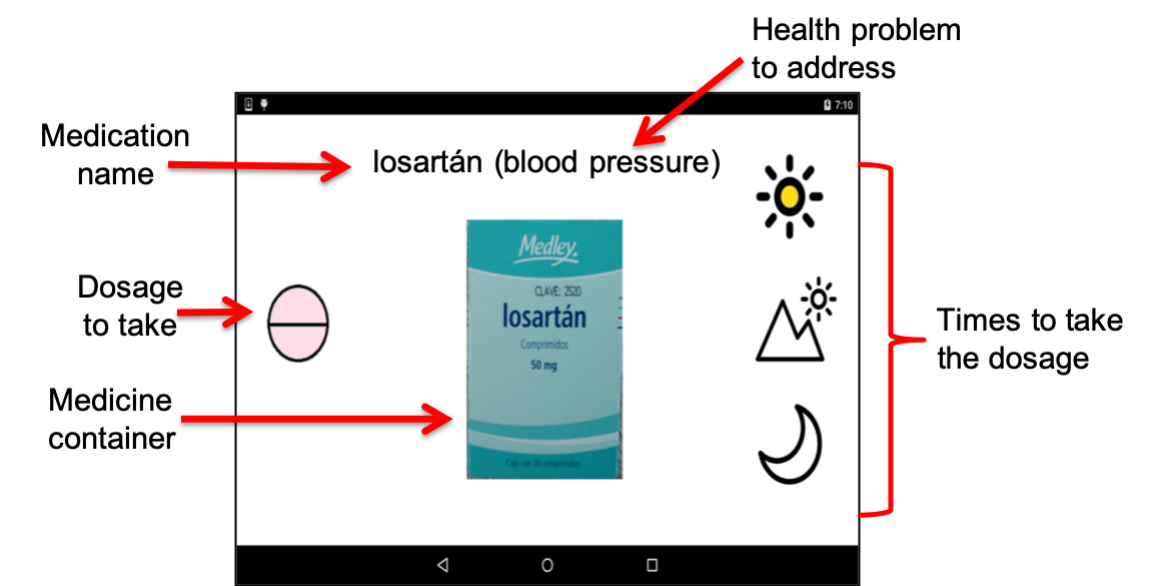
Medication Ambient Display reminding to medicate.

The parakeet provides auditory reminders (ie, parakeet whistle) and pictograms that inform how to take medications. For instance, Figure 5 denotes the morning doses (ie, 1 pill of Losartan for controlling blood pressure). On the right side, the pictogram depicts through representative icons that this medication has to be taken 3 times a day: morning, afternoon, and night. Moreover, the morning icon is colored to denote that the MAD reminder is for the first doses of the day.

#### Events to Enhance Older Adults’ Awareness of Whether the Medication Was Taken

**Figure 6:**
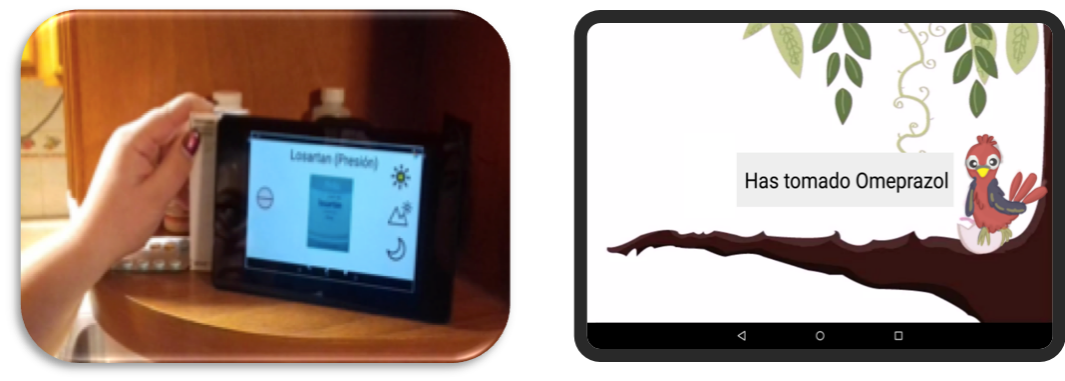
Registering the medication: After a senior medicates, she/he should move the corresponding medication container closer to the tablet in order for the attached NFC tag can be recognized by the tablet NFC reader (left); then, MAD acknowledges that the medicine was registered as taken on time (right).

These cues refer to actions performed by older adults to make taking medications more memorable [12]. As depicted in Figure 6, after an individual takes their medication, they move the tablet closer to the pill container to indicate that the medication was taken. We implemented this functionality through Near Field Communication (NFC) technology. Afterward, the parakeet acknowledges that the medication was registered as taken.

**Figure 7:**
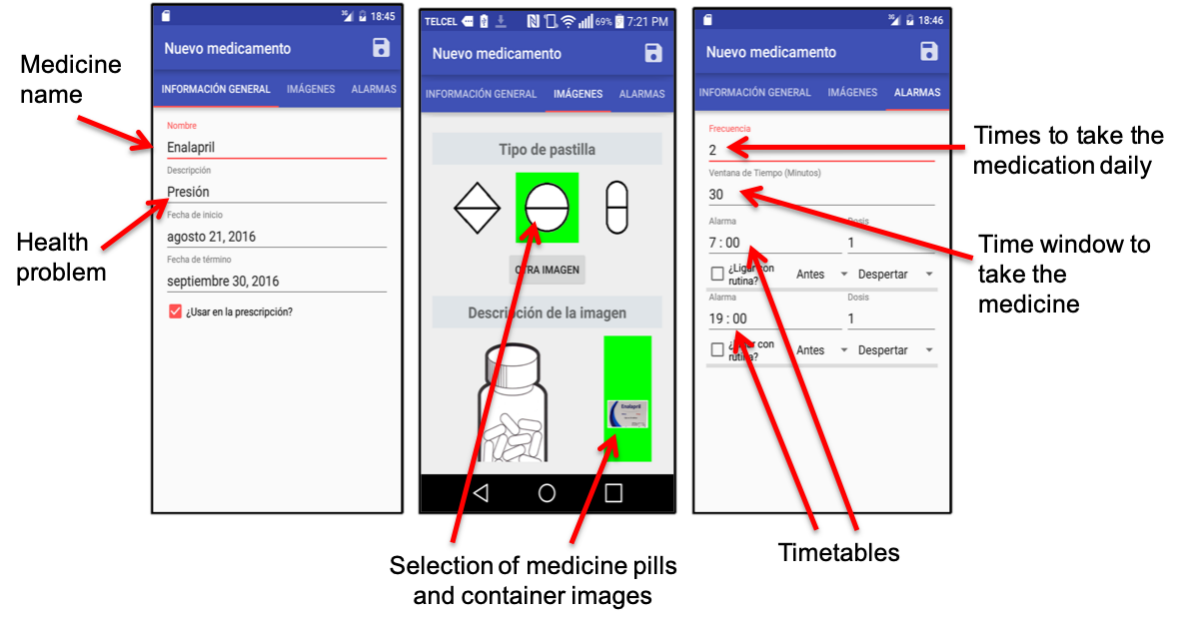
User interfaces of the administration component of Medication Ambient Display, which shows how it enabled the information registration of each medication.

We also implemented an administration component (see Figure 7), which we used to tailor MAD to participants’ medication prescriptions and the timetables that they reported to follow. As evaluating the ease of adapting and configuring MAD was out of the scope of this study, participants did not use the MAD administrator. Finally, MAD also includes a component to generate a log with the medication episodes registered by the participants through the NFC technology. A medication episode comprises the medication name and the corresponding timestamp. This information could be consulted during our visits to the seniors via this administrator component.

#### Intervention Procedure

We conducted a session with the research assistants, who made a random and blind allocation of the participants to the treatment group (TG) and the control group (CG). On the first day of this phase, research assistants visited older adults in the TG to introduce the MAD in the presence of caregivers by using the *spaced retrieval* approach, that is, teach, ask, wait, ask again, wait, and ask again [35]. This approach has been used to support the encoding, retention, and retrieval processes involved in interventions designed to assist medication taking [35]. Thus, we explained to participants how to carry out medication taking using the system. Afterward, we asked them to recall the system features we had just presented and assisted them as necessary. To do this, we activated each of the system’s functionalities and asked participants to use them (eg, interpreting a reminder, registering a medication, and consulting and interpreting their medication compliance). Then, we waited for 1 min and asked them to recall the system’s functionalities again. We asked them again 15 min later and repeated it once again. After the completion of the training session, which lasted 40 to 60 min approximately, the MAD was personalized according to the participant’s prescriptions and through discussions with the participant on an appropriate schedule for presenting the reminders. Research assistants used the MAD administrator to enter the medication names, health problems to address, timetables, and the frequency at which medications should be taken. Then, they attached and configured NFC tags to each of the pill containers, which included selecting the images that best represent the pills and their containers. Afterward, the MAD was placed in the area of participant’s home where they usually reported taking medications, mostly the kitchen, living room, and bedroom. The intervention phase lasted 5 weeks (see Figure 1), during which research assistants visited participants to collect data on medication adherence (from both the TG and the CG) and system usage (from the TG).

### Postintervention

After the intervention was completed, we removed the MAD from the participants’ homes. Research assistants then carried out weekly visits (weeks 16-17) to older adults from the TG to collect data that enabled us to understand how the withdrawal of the MAD affected their medication routine and adherence.

### Outcome Measures and Data Acquisition

Medication compliance (known as adherence as well) refers to “the act of conforming to the recommendations made by the provider concerning timing, dosage, and frequency of medication-taking” [27]. On the basis of this definition, we identified a set of variables as relevant for analyzing the effect of the external cues provided by the MAD on the participants’ medication adherence (see Table 2). Thus, these variables were used to address RQ1.

During the intervention phase, we also collected qualitative evidence about the system’s adoption, which enabled us to address RQ2. We interviewed the older adults in the TG regarding the system’s functionalities that they perceived as most useful, less useful, and the difficulties faced while using it. At the end of the postintervention stage, we interviewed participants to obtain their perceptions of how withdrawal from the MAD impacted their medication adherence. In addition, those caregivers who were at home during our visits were interviewed to obtain information on their involvement in the seniors’ medication activities. Our questions centered on the specific activities associated with the older adult’s medication regimen that caregivers were involved in and how they knew if the older adult took his or her pills in a given week. These semistructured interviews were administered by the first 3 authors of this paper.

### Data Analysis

We used Student *t* tests and chi-square tests to measure the statistical difference in age, the number of prescribed medicines, and self-reported medication adherence between the TG and the CG. A one-way repeated measure analysis of variance (ANOVA), dependent *t* tests, and independent *t* tests were used to find significant differences in medication adherence between the study phases and between the TG and the CG. The McNemar test was used to verify differences within the TG between the self-reported medication adherence in the recruitment and intervention phases. To determine whether any of the differences between the means estimated are statistically significant, we compared the *P* value with a significance level set to .05 [35].

For the qualitative analysis, we transcribed the collected data from their original Spanish version, that is, audio, handwritten notes, and photographs taken during the interviews. Individual quotes were translated into English for use in this paper. We followed the thematic analysis approach, which consists of generating initial codes from the data, searching for potential themes, contrasting the identified themes with the data, and iteratively refining them [36].

### Ethics Statement

The ethics review board of Faculty of Nursing approved the study protocol once we proposed how to address their suggestions on how to handle the withdrawal of the technology at the study end. We agreed to provide the participants of the TG with an adequate financial incentive that would allow them (if desired) to obtain a PC tablet similar to the one used during the study. Every week, participants received an economic incentive, approximately US $7 if they were in the CG and US $14 if they were in the TG. We obtained informed written consent from all individual participants.

## Results

### Baseline Characteristics of Study Participants

**Figure 8:**
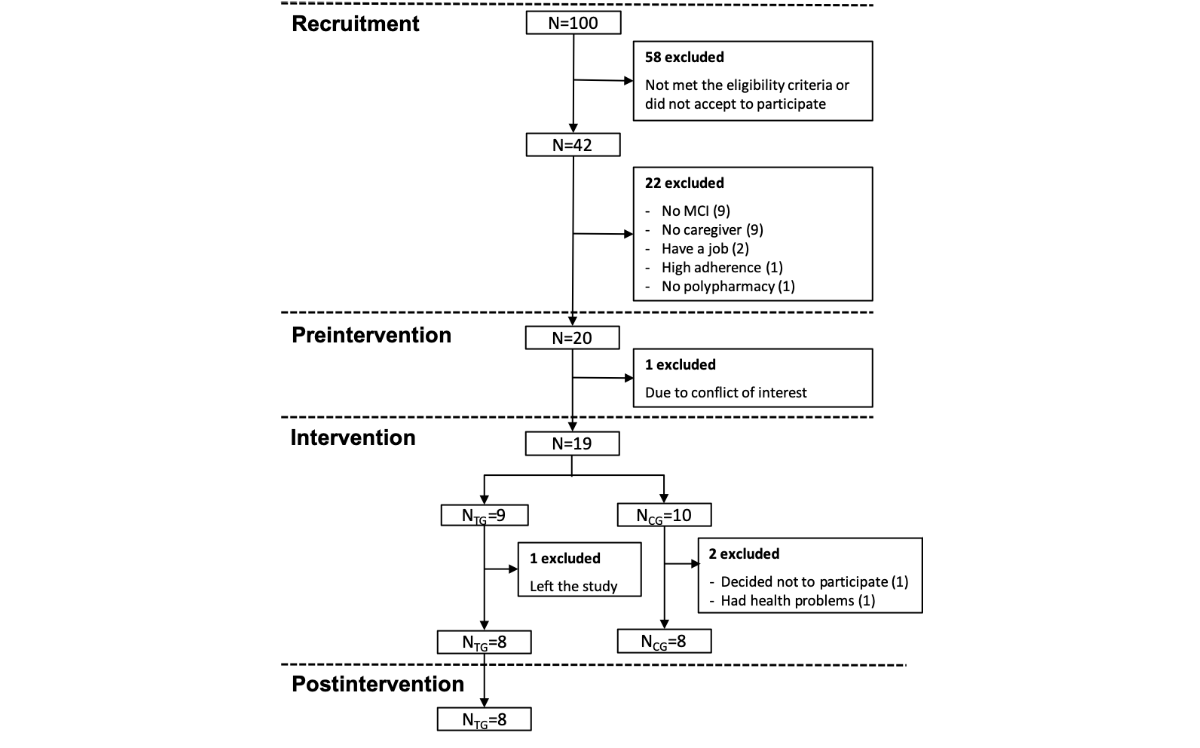
Flow diagram of the participants’ progress through the study phases. CG: control group; MCI: mild cognitive impairment; TG: treatment group.

The research assistants contacted approximately 100 older adults to participate in the study (see Figure 8). They identified 42 potential participants; 20 of them met the eligibility criteria and were enrolled in the study. However, only 16 completed the study. The analysis of baseline data presented in Table 3 indicated that the TG and the CG had no significant differences in age (*P*=.21), number of medicines taken (*P*=.33), self-reported medication adherence (*P*=.59), education in years (*P*=.35), gender (*P*=.25), relationship with caregiver (*P*=.57), and dosage outcome (*P*=.77).

**Table 3:** Characteristics of the participants.

Characteristic		Group		Statistics		
		Control	Treatment	*t* test (*df*)	Chi-square test (*df*)	*P* value
Age (years), mean (SD)		73.5 (8.3)	68.62 (6.2)	1.32 (15)	—^a^	.21
Education (years), mean (SD)		5.25 (3.8)	6.75 (2.1)	0.97 (15)	—	.35
Number of medications, mean (SD)		5.75 (1.8)	4.88 (1.6)	1.01 (15)	—	.33
Cognition		Mild^b^	Mild^b^	—	—	—
**Sex, n**					1.3 (1)	.25
	Female	5	7	—		
	Male	3	1	—		
**Caregiver relationship, n**					1.1 (1)	.57
	Spouse	3	3	—		
	Child	4	5	—		
	Other	1	0	—		
**Self-reported medication adherence, n**					0.2 (1)	.59
	Low	2	3	—		
	Middle	6	5	—		
**Pill counting medication adherence (%), mean (SD)**					0.30 (15)	.77
	Baseline	79.87 (17.9)	80.9 (16)	—		

^a^Not applicable.

^b^Cognitive impairment was assessed with the Short Portable Mental Status Questionnaire because it is appropriate for low-literacy persons [26].

### Adherence to Medication (Research Question 1)

Although underadherence was the most predominant behavior, overadherence was also found during the stages of different studies, for both the TG and the CG (see Multimedia Appendix 1). We identified overmedication and undermedication during the intervention phase not only according to the pill counting technique but also according to the medication episodes recorded in the MAD’s log. For instance, Multimedia Appendix 1 shows that participant P2 has adherence rates higher than 100% according to both techniques. Similarly, overmedication could be present in the baseline data, which impacted the high medication rates registered for some older adults (eg, participants P7 and P8). However, our results show that the introduction of the MAD’s external cues to the TG resulted in significant improvements in the average rates of dosage outcomes, which addresses RQ1.

#### Dosage_pill_ (Treatment Group)

The TG improved their adherence behavior (dosage), increasing from 80.9% in the preintervention phase to 95.97% in the intervention phase. However, it decreased to 76.71% in the postintervention phase. Using a one-way repeated measures ANOVA, we compared the effect of the MAD in the TG during the 3 phases. It showed a significant statistical difference between at least two of the phases (*F*_2,14_=6.59; *P*=.0096). With a post hoc analysis using the Tukey honestly significant difference test, we identified a statistical difference between the preintervention and intervention phases (*P*=.02) and between the intervention and postintervention phases (*P*=.002); Cohen effect size values (*d*=1.35 and *d*=1.72, respectively) suggest a high practical significance in both cases. In addition, there was no statistical difference between the preintervention and the postintervention phases (*P*=.73). Therefore, these results are evidence that the external cues of the MAD contributed to improving the medication intake behaviors of older adults.

#### Dosage_pill_ (Control Group)

The CG increased their medication adherence between the preintervention (mean 79.87% [SD 20.49%]) and intervention phases (mean 88.18% [SD 20.49%]). However, according to a paired-samples *t* test, there was not a significant difference (*t*_7_=1.15; *P*=.14). The Cohen effect size value (*d*=0.41) suggests a medium practical significance. We attributed this increase in dosage rates to the medication monitoring conducted weekly by the research assistants and to the basket provided to organize the pills containers.

#### Dosage_pill_ (Treatment Group vs Control Group)

The medication adherence rate of the TG (mean 95.97% [SD 6.08%]) was higher than that of the CG (mean 88.18% [SD 13.06%]) during the intervention phase. However, according to a independent samples *t* test, there was not a significant difference (*t*_14_=1.53; *P*=.08). The Cohen effect size value (*d*=0.76) suggests a high practical significance. The monitoring conducted by research assistants impacted both groups.

#### Dosage_pill_ Versus Dosage_MAD_ (Treatment Group)

A one-way ANOVA was conducted to compare the effect of *Dosage_pill_* versus *Dosage_MAD_* on the adherence of the TG. An ANOVA showed that the effect of both measuring instruments on adherence was not significant (*F*_1,14_=0.21; *P*=.65). This confirms the suitability of the results obtained through the pill counting technique to explain how the MAD supports adherence to medication. In addition, as described in the following results, *Dosage_MAD_* estimation enabled us to determine how the MAD reminders helped older adults take their medications at the prescribed times.

#### Timely and Reminder Dependency Rates

We obtained 2224 medication episodes registered in the MAD’s log, of which 93.17% (2072/2224) were registered as taken on time, and 88.35% (1830/2224) of these timely episodes were taken after the MAD reminders. As illustrated in Table 4, most of the participants showed a high reminder dependency rate. These results are evidence that the MAD reminders resulted in medication-taking behaviors consistently.

**Table 4:** Timely and reminder dependency rates estimated by participants.

Participants in the treatment group	Medication episodes rate (%)	Timely episodes rate (%)	Reminder dependency rate (%)
P1	86.46	87.37	93.64
P2	100.67	92	94.2
P3	93.83	94.92	100
P4	100	92.44	32.19
P5	94.71	97.21	98.56
P6	99.23	89.92	93.97
P7	98.56	98.06	99.5
P8	100.43	93.16	98.62

#### Self-Reported Medication Adherence

We found that MAQ-8 scores and *Dosage_pill_* outcomes are not correlated but are independent of each other (N=16; ρ=−0.29; *P*=.27). A McNemar test determined that there was no statistically significant difference in the MAQ-8 scores obtained in the preintervention and postintervention stages (*P*=.25). These results suggest that using the MAD did not change older adults’ perception about their medication adherence.

### System Adoption (Research Question 2)

The collected qualitative evidence helps us to address RQ2 and to complement the quantitative results. We analyzed the data collected from the interviews administered to participants P1 to P8 of the TG, and we discovered the findings described in the following subsections.

#### Usability of the Ambient Modalities That Implement the Medication Ambient Display’s External Cues

All the older adults provided answers that lead us to conclude that the most useful cues were the auditory reminders, followed by the stylized representation of medication adherence (eg, parakeet growth). Some participants explained how these external cues caught their attention so that they can medicate themselves. Participant P6 said:

Sometimes I am busy or just thinking about something else, and I forgot what I have to do; but now when the parakeet sings and sings, it reminds me to medicate.

This finding is supported by the high rates in timely and reminder dependency measures. For instance, participant P1 improved her medication-taking behavior because she developed a high dependency on the ambient reminders (see Table 4), which affected her medication compliance during the postintervention phase (see Multimedia Appendix 1). She reported:

When I heard the parakeet whistle, I came to the kitchen to take my medicines...It was better when I had the system.

All older adults, except participant P4, reported that they did not consult the detailed information on their medication adherence, but they verified if the parakeet grew after registering the medication as taken. Thus, participant P4 was the only older adult who did not develop a high reminder dependency rate (see Table 4) to stabilize her medication adherence (see Multimedia Appendix 1), but she relied on the stylized abstract representations and detailed report of her adherence; in addition, she expressed interest in consulting her medication adherence from the last week or month.

#### Older Adults’ Perception of Their Medication Adherence

Although adverse drug events associated with overmedication and undermedication were identified, older adults hardly recognized that before using the MAD; there were times when they might have forgotten to take a medication. For instance, although participant P4 explicitly stated that she did not forget to take her pills, her husband contradicted her. Participant P6 was the only participant who, during the interviews, admitted forgetting to take her medication because as a consequence, she had symptoms of her disease:

[Before using the MAD] I realized that I had forgotten to take the [night doses] pill for my blood pressure, until the next morning my head hurt and I felt dizzy.

On the other hand, we have no evidence that older adults were aware that they sometimes overmedicate because they forgot that they had taken their medication.

#### Integration of Medication Routine Into Daily Activities

Older adults intend to take their medications when carrying out some of their daily routines. We identified that some older adults fail to associate medication with their daily activities effectively. For instance, participant P3 indicated:

I plan to take my night medication before going to sleep, but before using MAD, sometimes I realized that I had forgotten to medicate until I was in bed, and murmured: “Ay! I have not taken the pill.” However, now, it [MAD] sounds, I take my medication, and then, I go to sleep.

On the other hand, we found that leaving home or traveling affected the use of the MAD and probably the medication adherence of participants. For instance, several participants (P2, P3, and P6) reported situations in which they could not register a medication intake timely because they had to leave home. For instance, participant P6 reported:

I did not take my medication this Wednesday since I had to leave the house urgently, but I took it later.

Also, only 1 participant (P4) felt confident enough to take the system with her when she left home.

#### External Cues Mediated Caregivers’ Assistance

The family caregivers commonly served as the main support actor and assisted with managing the seniors’ medications. Before using the MAD, caregivers tended to be aware of the medication timetables to remind older adults or ask older adults if they had medicated. We observed that the external cues of the MAD acted as triggers that facilitated caregivers’ assistance. That is, the cues did not overwhelm family members but were an appropriate mediation strategy to support seniors’ medication routines. For instance, the husband of participant P4 perceived that the MAD enabled him to be aware when she took the correct medications. For an adolescent caregiver, the system enabled him to feel less worried about having to remind her grandmother (participant P1) to medicate:

If I have to do my homework, I can focus on doing it.

The MAD also helped to assure caregivers that older adults would not forget to take their medications; for example, participant P6 said:

My children used to forget reminding me to medicate [before using the MAD]. Currently, they hear the parakeet, and then they make sure if I took them.

## Discussion

### Principal Findings

Our quantitative results show that providing the external cues supported by the MAD resulted in significant improvements in the average rates of dosage outcomes for older adults. This is because the ambient modalities used for implementing these external cues were useful for drawing the attention of older adults. We found that external cues (1) reminded them to take medications, (2) enabled them to recognize if a medication was recorded as taken, and (3) provided immediate awareness about how they followed their medication regimens.

We learned that providing older adults with an abstract and stylized representation of their medication adherence, which could be peripherally perceived, was better accepted than medication adherence reports that need to be explicitly evoked. However, this stylized and abstract modality of representation was not enough to make participants aware of their medication problems related to undermedication and overmedication. Previous research has demonstrated that feedback-based systems that are consulted explicitly and daily help seniors identify their medication errors and then self-regulate their medication behavior [20]. We consider that providing timely detailed information about medication adherence may help older adults perceive the usefulness of systems designed to aid medication uptake and, therefore, encourage their adoption. From our study, we learned that it is necessary to make the feedback more salient to provide them with sufficient knowledge of their medication problems.

Similar to our results, other studies have shown that when older adults stop using medication aiding systems, their medication adherence is affected [20]. Our participants showed high dependence on the MAD reminders; therefore, when the system was removed from their homes, their adherence to medications was negatively affected. We also found that older adults tend to link their medication to other daily tasks [1,37], for example, take medication before going to sleep. We conclude that ambient displays should be flexible enough to adapt external cues according to the activities that older adults associate with their medication intake behaviors. These include taking into account the daily routines in which the medication should be inserted (eg, before meals), in addition to taking into account context changes (eg, going out from home). We hypothesized that including adaptation mechanisms to the MAD that allow seniors to configure external cues to remind them to take medications in a specific context instead of a specific window of time would allow that medication to be integrated into their daily routine. For instance, if an older adult proposes to take their medications when preparing his or her coffee in the morning, the MAD could include cues to help them remember to associate their medication with that activity. In this case, the MAD would be training older adults during a period to take their medication when that specific context arises. We hypothesized that supporting this strategy may reduce the dependency of older adults to the MAD’s medication reminders, as it may help them develop the habit of taking their medication in the same context consistently.

Furthermore, we identified that external cues of the MAD provided caregivers with better awareness of older adults’ medication adherence. This awareness was 2-fold: (1) when auditory reminders were perceived by caregivers, they made sure that the reminders reached the target recipients, and (2) medication adherence representations enabled caregivers to be aware of medications taken. Therefore, providing external cues through ambient displays helps family caregivers to better support seniors to follow their medication regimens.

### Limitations

#### Economic Incentive for Participating

The use of financial incentives has been questioned because they may provide inducements to participate in a study for financial purposes only, and vulnerable populations are prone to be enticed by the financial reward and be more willing to accept any study risks [38]. In our opinion, offering an incentive facilitated recruitment of participants and allowed us to access their data, which would otherwise be considered an obtrusive task. For instance, 2 participants explicitly asked for the weekly economic incentive to allow the research assistants to collect data; another participant questioned the incentive since she considered it was unnecessary for being part of the study. Although we are not able to conclude on how the incentive impacted the adoption of the technology, our findings indicate that the MAD was accepted not only by older adults but also by their family caregivers.

#### Setting Characteristics

We observed that some participants faced problems in managing their medications, such as accumulating medications, confusing medications because they look alike, and tending to give medications to others, which may not be an appropriate practice. The design of our study was limited in that the MAD system was personalized according to the prescribed medication regimens and timetables that the participants followed. Although using our technology did not introduce any risk, it might have supported inappropriate medication routines adopted by older adults to overcome some of the barriers imposed by the setting. We recognize the importance of conducting a contextual study before conducting a technology evaluation. The contextual study should be designed in collaboration with clinical or nursing specialists to reduce the complexity of older adults’ medication regimens and the risks associated with the way they manage medications. Finally, we are not able to state that our results are generalizable to the whole Mexican elderly population. This is because participants were primarily from a low-income socioeconomic stratum; therefore, exploring this technology among high-income elders who have access to private health care services might produce different findings.

#### Study Methodology

The pill counting technique can be prone to human error. So, one limitation of this study is that we were not able to identify which overmedication and undermedication events were registered by the research assistants erroneously. Using electronic monitoring devices (EMDs), such as the Medication Event Monitoring System, could have overcome this limitation to some extent, although using EMDs does not guarantee that a person has taken their medication [39]. Moreover, the cost of EMDs and the logistics of integrating them within clinical protocols may be limiting factors to their adoption for research in clinical contexts [39]. One possible solution to overcome this limitation could be to combine data collection techniques.

#### Study Duration

Another limitation of the study is its duration time. Some research works have identified that older adults should have an adaptation period to an intervention, and after a specific period of using it, reliable data can be collected to measure its effectiveness for improving medication compliance [3]. We provided evidence that our study enabled us to get an understanding of the feasibility of the MAD to improve adherence to medication. However, extending the time of both stages, intervention and postintervention, would have allowed us to understand the efficacy of the MAD to sustain high rates of medication adherence.

### Comparison With Prior Studies

Table 5 shows an overview of some studies published in the last decade, which were conducted to assess technological-based interventions to support behavioral strategies to improve older adults’ medication adherence, such as providing reminders [18,19,40-43], medication feedback [20,39-41], and self-monitoring of pills [20,39,42]. Some of these studies also included a patient education strategy [15,42,43], which is considered a traditional approach used to tackle the medication adherence problem [44]. The technologies evaluated in these studies included mobile phones, tablets, EMDs, and pill dispensers. These studies have assessed the technologies’ effect on medication adherence, in addition to evaluating older adults’ acceptance. Most of them used both subjective (eg, self-reporting) and objective (eg, pill counting and system logs) adherence assessment methods [15,20,40-43] because no single method is sufficiently reliable and accurate [30]. Nonetheless, some of these studies are limited to assessing only the adherence to medications taken for a particular health problem [40,41] and did not take into account older adults with multiple morbidities and polypharmacy [15,39,40], which are factors that contribute to increasing the risk of nonadherence [45,46]. Other reasons for nonadherence include low literacy, cultural factors, and inadequate social support [45]. In this sense, none of the studies presented in Table 5 examined the involvement of family caregivers, although some of them report that their technologies offered mechanisms to enable the family caregivers’ participation [19,39-41]. In contrast, our study assessed the effect of our approach by using objective medication adherence measures; in addition, we obtained qualitative findings that explained the adoption of the MAD from the perspectives of both seniors and their relatives.

**Table 5:** Overview of some studies to assess different technological approaches to support older adults’ medication adherence.

Data extracted from the studies		Morawski et al [40]	Mertens et al [41]	Robiner et al [39]	Grindrod et al [18]	Park et al [15]	Lee and Dey [20]	Perera et al [42]	Patel et al [43]	Reeder et al [19]	De Oliveira et al [21]
**Technology**											
	Mobile phone apps	X^a^	—^b^	—	—	—	—	X	X	—	X
	SMS	—	—	—	—	X	—	—	—	—	—
	Tablet	—	X	—	X	—	X	—	—	—	—
	Dispenser	—	—	—	—	—	—	—	—	X	—
	Monitoring	—	—	X	—	—	X	—	—	—	—
**Functions**											
	Remind	X	X	—	X	—	—	X	X	X	—
	Register taken doses	X	X	—	—	—	—	X	X	X	X
	Educate	—	—	—	—	X	—	X	X	—	—
	Feedback	X	X	X	—	—	X	—	—	—	—
	Caregiver participation	X	X	X	—	—	—	—	—	X	—
	Social game	—	—	—	—	—	—	—	—	—	X
**Evaluation**											
	Total participants (older adults)	413	24	6	35	90	12	28	48	96	16
	Medicines	2_Hipertension_	+3_cardvascular desease_	1_renal_	+1_several_	2_cardiovascular desease_	N/S^c^	3_HIV_	3_Hipertension_	+11_several_	+1_serveral_
	Methods to measure adherence: subjective and objective	Subjective and objective	Subjective and objective	Objective	—	Subjective and objective	Subjective and objective	Subjective and objective	Subjective and objective	—	—
	Acceptance	—	X	X	X	X	—	X	X	X	X

^a^X: studies that assessed the acceptance of the system by the participants.

^b^Not applicable.

^c^N/S: nonsignificant.

### Conclusions

The external cues provided by our ambient display not only improved the medication adherence of the elderly but also encouraged caregiver involvement. We found that the external cues perceived as most useful were those that reminded participants to take medications, helped seniors recognize if medications were recorded as taken, and provided immediate and abstract representations of their medication adherence. We identified that external cues did not overwhelm family members but were an appropriate mediation strategy to support older adults’ medication routines. We also recognized the potential of providing external cues to enable older adults to associate medication routines with their daily routines appropriately. For future work, we plan to conduct studies to assess the feasibility of external ambient cues to support the seamless integration of medication regimens into the daily routines of the elderly.

## Appendix

Multimedia Appendix 1 Individual dosage rates estimated through the pill counting technique (Dosagepill) and the medication episodes recorded in MAD’s log (DosageMAD). MAD: Medication Ambient Display.

Multimedia Appendix 2 CONSORT-EHEALTH checklist (V 1.6.1).

## Abbreviations

ANOVA: analysis of variance
CG: control group
CONACyT: National Council of Science and Technology
EMD: electronic monitoring device
IMSS: Mexican Institute of Social Security
MAD: Medication Ambient Display
MAQ-8: 8-item Medication Adherence Questionnaire
MedMaIDE: Medication Management Instrument for Deficiencies in the Elderly
NFC: Near Field Communication
RQ: research question
TG: treatment group
UNICOM: Community Center of the University

## References

[1] Insel KC, Einstein GO, Morrow DG, Hepworth JT (2013). A multifaceted prospective memory intervention to improve medication adherence: design of a randomized control trial. Contemp Clin Trials.

[2] Murray MD, Morrow DG, Weiner M, Clark DO, Tu W, Deer MM, Brater DC, Weinberger M (2004). A conceptual framework to study medication adherence in older adults. Am J Geriatr Pharmacother.

[3] Insel KC, Einstein GO, Morrow DG, Koerner KM, Hepworth JT (2016). Multifaceted prospective memory intervention to improve medication adherence. J Am Geriatr Soc.

[4] Gillespie R, Mullan J, Harrison L (2014). Managing medications: the role of informal caregivers of older adults and people living with dementia. A review of the literature. J Clin Nurs.

[5] Kang HS, Myung W, Na DL, Kim SY, Lee J, Han S, Choi SH, Kim S, Kim S, Kim DK (2014). Factors associated with caregiver burden in patients with Alzheimer's disease. Psychiatry Investig.

[6] Smith G, Della Sala S, Logie RH, Maylor EA (2000). Prospective and retrospective memory in normal ageing and dementia: a questionnaire study. Memory.

[7] Gillespie RJ, Harrison L, Mullan J (2015). Medication management concerns of ethnic minority family caregivers of people living with dementia. Dementia (London).

[8] Lingler JH, Sereika SM, Amspaugh CM, Arida JA, Happ ME, Houze MP, Kaufman RR, Knox ML, Tamres LK, Tang F, Erlen JA (2016). An intervention to maximize medication management by caregivers of persons with memory loss: Intervention overview and two-month outcomes. Geriatr Nurs.

[9] While C, Duane F, Beanland C, Koch S (2013). Medication management: the perspectives of people with dementia and family carers. Dementia (London).

[10] Cramer JA (1998). Enhancing patient compliance in the elderly. Role of packaging aids and monitoring. Drugs Aging.

[11] García-Vázquez JP, Rodríguez MD, Andrade AG, Bravo J (2011). Supporting the strategies to improve elders’ medication compliance by providing ambient aids. Pers Ubiquit Comput.

[12] Rodríguez M, Zárate E, Stawarz K, García-Vázquez J, Ibarra E (2015). Ambient computing to support the association of contextual cues with medication taking. Rev Mex Ing Bioméd.

[13] Zárate-Bravo E, García-Vázquez JP, Rodríguez MD, Serino S, Matic A, Giakoumis D, Lopez G, Cipresso P (2016). An ambient medication display to heighten the peace of mind of family caregivers of older adults: A study of feasibility. Pervasive Computing Paradigms for Mental Health. MindCare 2015. Communications in Computer and Information Science. Volume 604.

[14] Cramer JA, Roy A, Burrell A, Fairchild CJ, Fuldeore MJ, Ollendorf DA, Wong PK (2008). Medication compliance and persistence: terminology and definitions. Value Health.

[15] Park LG, Howie-Esquivel J, Chung ML, Dracup K (2014). A text messaging intervention to promote medication adherence for patients with coronary heart disease: a randomized controlled trial. Patient Educ Couns.

[16] Park LG, Howie-Esquivel J, Dracup K (2014). A quantitative systematic review of the efficacy of mobile phone interventions to improve medication adherence. J Adv Nurs.

[17] Stawarz K, Cox L, Blandford A (2014). Don't Forget Your Pill!: Designing Effective Medication Reminder Apps That Support Users' Daily Routines. Proceedings of the SIGCHI Conference on Human Factors in Computing Systems.

[18] Grindrod KA, Li M, Gates A (2014). Evaluating user perceptions of mobile medication management applications with older adults: a usability study. JMIR Mhealth Uhealth.

[19] Reeder B, Demiris G, Marek KD (2013). Older adults' satisfaction with a medication dispensing device in home care. Inform Health Soc Care.

[20] Lee M, Dey A (2014). Real-Time Feedback for Improving Medication Taking. Proceedings of the SIGCHI Conference on Human Factors in Computing Systems.

[21] de Oliveira R, Cherubini M, Oliver N (2010). MoviPill: Improving Medication Compliance for Elders Using a Mobile Persuasive Social Game. Proceedings of the 12th ACM international conference on Ubiquitous computing.

[22] Tschanz M, Dorner Tl, Holm J, Denecke K (2018). Using eMMA to manage medication. Computer.

[23] Lobo J, Ferreira L, Ferreira A (2017). CARMIE: A conversational medication assistant for heart failure. J E-Health Med Commun.

[24] Thabane L, Ma J, Chu R, Cheng J, Ismaila A, Rios LP, Robson R, Thabane M, Giangregorio L, Goldsmith CH (2010). A tutorial on pilot studies: the what, why and how. BMC Med Res Methodol.

[25] Eysenbach G, CONSORT-EHEALTH Group (2011). CONSORT-EHEALTH: improving and standardizing evaluation reports of Web-based and mobile health interventions. J Med Internet Res.

[26] Iglesiaa J, Dueñas R, Vilchesa M, Tabernéa C, Colomerc C, Luquec R (2001). Adaptación y validación al castellano del cuestionario de Pfeiffer (SPMSQ) para detectar la existencia de deterioro cognitivo en personas mayores e 65 años. Medicina Clínica.

[27] Morisky DE, Green LW, Levine DM (1986). Concurrent and predictive validity of a self-reported measure of medication adherence. Med Care.

[28] Lavsa SM, Holzworth A, Ansani NT (2011). Selection of a validated scale for measuring medication adherence. J Am Pharm Assoc (2003).

[29] Orwig D, Brandt N, Gruber-Baldini AL (2006). Medication management assessment for older adults in the community. Gerontologist.

[30] Giardini A, Martin MT, Cahir C, Lehane E, Menditto E, Strano M, Pecorelli S, Monaco A, Marengoni A (2016). Toward appropriate criteria in medication adherence assessment in older persons: Position Paper. Aging Clin Exp Res.

[31] Wirtz VJ, Reich MR, Flores RL, Dreser A (2008). Medicines in Mexico, 1990-2004: systematic review of research on access and use. Salud Publica Mex.

[32] Mahmood A, Elnour AA, Ali AA, Hassan NA, Shehab A, Bhagavathula AS (2016). Evaluation of rational use of medicines (RUM) in four government hospitals in UAE. Saudi Pharm J.

[33] Mao W, Vu H, Xie Z, Chen W, Tang S (2015). Systematic review on irrational use of medicines in China and Vietnam. PLoS One.

[34] Yen P, Bakken S (2012). Review of health information technology usability study methodologies. J Am Med Inform Assoc.

[35] Montgomery DC (2012). Design And Analysis Of Experiments. Eighth Edition.

[36] Braun V, Clarke V (2006). Using thematic analysis in psychology. Qual Res Psychol.

[37] Stawarz K, Rodríguez MD, Cox AL, Blandford A (2016). Understanding the use of contextual cues: design implications for medication adherence technologies that support remembering. Digit Health.

[38] Groth SW (2010). Honorarium or coercion: use of incentives for participants in clinical research. J N Y State Nurses Assoc.

[39] Robiner WN, Flaherty N, Fossum TA, Nevins TE (2015). Desirability and feasibility of wireless electronic monitoring of medications in clinical trials. Transl Behav Med.

[40] Morawski K, Ghazinouri R, Krumme A, McDonough J, Durfee E, Oley L, Mohta N, Juusola J, Choudhry NK (2017). Rationale and design of the Medication adherence Improvement Support App For Engagement-Blood Pressure (MedISAFE-BP) trial. Am Heart J.

[41] Mertens A, Brandl C, Miron-Shatz T, Schlick C, Neumann T, Kribben A, Meister S, Diamantidis CJ, Albrecht U, Horn P, Becker S (2016). A mobile application improves therapy-adherence rates in elderly patients undergoing rehabilitation: A crossover design study comparing documentation via iPad with paper-based control. Medicine (Baltimore).

[42] Perera AI, Thomas MG, Moore JO, Faasse K, Petrie KJ (2014). Effect of a smartphone application incorporating personalized health-related imagery on adherence to antiretroviral therapy: a randomized clinical trial. AIDS Patient Care STDS.

[43] Patel S, Jacobus-Kantor L, Marshall L, Ritchie C, Kaplinski M, Khurana PS, Katz RJ (2013). Mobilizing your medications: an automated medication reminder application for mobile phones and hypertension medication adherence in a high-risk urban population. J Diabetes Sci Technol.

[44] Banning M (2009). A review of interventions used to improve adherence to medication in older people. Int J Nurs Stud.

[45] Williams A, Manias E, Walker R (2008). Interventions to improve medication adherence in people with multiple chronic conditions: a systematic review. J Adv Nurs.

[46] Claxton AJ, Cramer J, Pierce C (2001). A systematic review of the associations between dose regimens and medication compliance. Clin Ther.

