# Supplementary figures and images for: Supporting the Medication Adherence of Older Mexican Adults Through External Cues Provided With Ambient Displays: Feasibility Randomized Controlled Trial
Source: JMIR Mhealth Uhealth. 2020 Mar 2;8(3):e14680. doi: 10.2196/14680 (PMC7076413; doi:10.2196/14680)

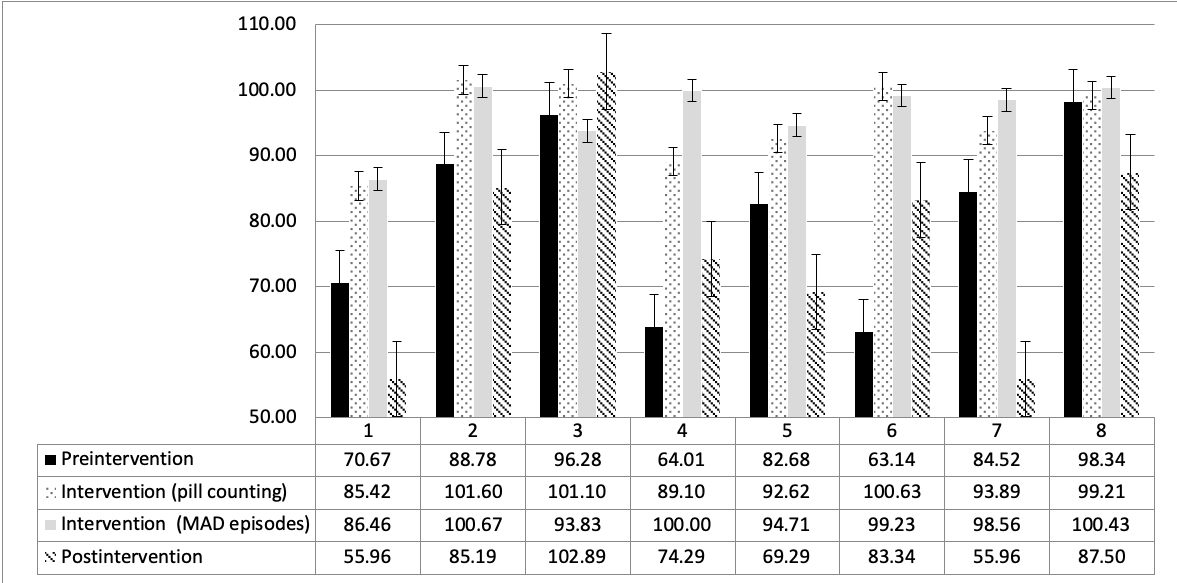

Supplement: Multimedia Appendix 1 [file mhealth_v8i3e14680_app1.png]
